# Update: Influenza Activity — United States, September 30, 2012–February 9, 2013

**Published:** 2013-02-22

**Authors:** World Health Organization Collaborating Center for Surveillance, Epidemiology, and Control of Influenza, Lenee Blanton, Scott Epperson, Lynnette Brammer, Krista Kniss, Desiree Mustaquim, Craig Steffens, Alejandro Perez, Sandra S. Chaves, Teresa Wallis, Julie Villanueva, Xiyan Xu, Lyn Finelli, Anwar Isa Abd Elal, Larisa Gubareva, Joseph Bresee, Alexander Klimov, Nancy Cox

**Affiliations:** Influenza Div, National Center for Immunization and Respiratory Diseases, CDC

Influenza activity in the United States began to increase in mid-November and remained elevated through February 9, 2013. During that time, influenza A (H3N2) viruses predominated overall, followed by influenza B viruses. This report summarizes U.S. influenza activity* since the beginning of the 2012–13 influenza season and updates the previous summary (1).

## Viral Surveillance

**FIGURE 1:**
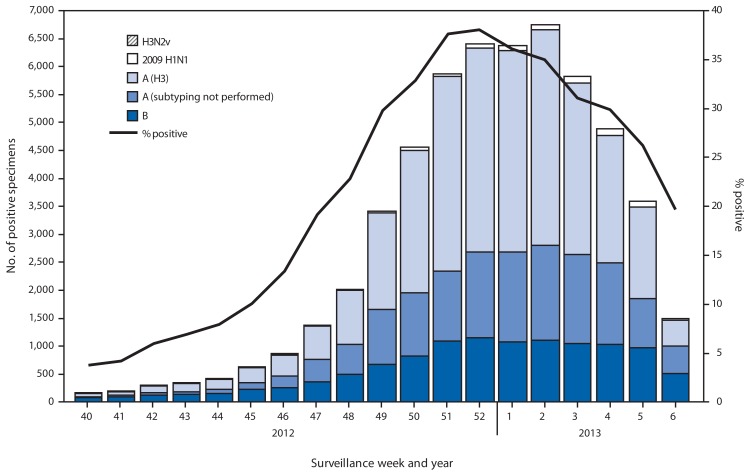
Number and percentage of respiratory specimens testing positive for influenza, by type, surveillance week, and year — U.S. World Health Organization and National Respiratory and Enteric Virus Surveillance System collaborating laboratories, United States, 2012–13 influenza season

During September 30, 2012–February 9, 2013, approximately 140 World Health Organization (WHO) and National Respiratory and Enteric Virus Surveillance System collaborating laboratories in the United States tested 203,706 respiratory specimens for influenza viruses; 55,470 (27.2%) were positive (Figure 1). Of these, 44,035 (79%) were influenza A viruses, and 11,435 (21%) were influenza B viruses. Of the 44,035 influenza A viruses, 29,914 (68%) were subtyped; 29,091 (97%) of these were influenza A (H3) viruses, and 823 (3%) were influenza A (H1N1)pdm09 (pH1N1) viruses. The percentage of specimens testing positive for influenza increased through the week ending December 29, 2012 (week 52), when 38.1% tested positive, and decreased subsequently. In the week ending February 9, 2013 (week 6), 19.7% of specimens tested positive. Since the start of the influenza season to February 9, 2013, influenza A (H3) viruses predominated in the United States overall, followed by influenza B viruses, while pH1N1 viruses were identified less frequently.

## Novel Influenza A Viruses

One infection with an influenza A (H3N2) variant virus (H3N2v) was reported to CDC during the week ending December 8, 2012 (week 49) from Minnesota. Close contact between the patient and swine in the week preceding illness was reported. The patient fully recovered, and no further cases were identified in contacts of the patient. This is the second H3N2v infection reported for the 2012–13 influenza season (1).

## Antigenic Characterization

WHO collaborating laboratories in the United States are requested to submit a subset of their influenza-positive respiratory specimens to CDC for further antigenic characterization. CDC has antigenically characterized 1,088 influenza viruses collected during the 2012–13 season, including 86 pH1N1, 677 influenza A (H3N2), and 325 influenza B viruses. All pH1N1 viruses were characterized as A/California/7/2009-like (H1N1), which is the 2012–13 influenza A (H1N1) component of the 2012–13 Northern Hemisphere vaccine. A total of 673 (99.4%) of the 677 influenza A (H3N2) viruses were characterized as A/Victoria/361/2011-like (H3N2), the influenza A (H3N2) component of the 2012–13 Northern Hemisphere vaccine. Of the 325 influenza B viruses tested, 230 (71%) belong to the B/Yamagata lineage and were characterized as B/Wisconsin/1/2010-like, the influenza B component of the 2012–13 Northern Hemisphere vaccine; 95 (29%) of the influenza B viruses tested belong to the B/Victoria lineage of viruses.

## Antiviral Resistance of Influenza Virus Isolates

Since October 1, 2012, a total of 1,702 influenza viruses have been tested for resistance to influenza antiviral medications. None of the 1,072 influenza A (H3N2) or the 396 influenza B viruses was resistant to either oseltamivir or zanamivir. Among 234 pH1N1 viruses tested for resistance to oseltamivir, two (0.9%) were found to be resistant, and of the 97 viruses tested for resistance to zanamivir, none were found to be resistant, including one of the two oseltamivir-resistant pH1N1 viruses. Additional laboratory testing, including testing for resistance to zanamvir, is pending on the second oseltamivir-resistant pH1N1 virus. High levels of resistance to the adamantanes persist among pH1N1 and influenza A (H3N2) viruses.

## Outpatient Illness Surveillance

**FIGURE 2:**
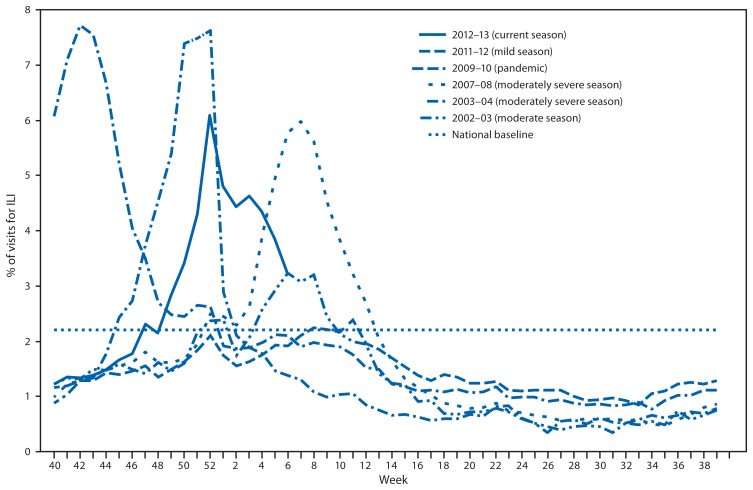
Percentage of visits for influenza-like illness (ILI) reported by the U.S. Outpatient Influenza-Like Illness Surveillance Network (ILINet), by surveillance week and year — United States, 2012–13 and selected previous influenza seasons* * Data as of February 16, 2013.

Since September 30, 2012, the weekly percentage of outpatient visits for influenza-like illness (ILI)† reported by approximately 1,900 U.S. Outpatient ILI Surveillance Network (ILINet) providers in 50 states, New York City, Chicago, the U.S. Virgin Islands, and the District of Columbia that comprise ILINet, has ranged from 1.2% to 6.1%. From the week ending November 24, 2012 (week 47) to February 9, 2013 (week 6), the percentage equaled or exceeded the national baseline§ of 2.2% for 12 consecutive weeks (Figure 2). During the 1997–98 through 2011–12 seasons, peak weekly percentages of outpatient visits for ILI ranged from 2.4% to 7.7% and remained above baseline levels for an average of 12 weeks (range: 1–18 weeks). For the week ending February 9, 2013 (week 6), all 10 U.S. Department of Health and Human Services regions¶ continued to report ILI activity above region-specific baseline levels.

Data collected in ILINet are used to produce a measure of ILI activity** by jurisdiction. During the week ending February 9, 2013 (week 6), 11 states and New York City experienced high ILI activity (Alabama, California, Idaho, Kansas, Michigan, Missouri, Nevada, New Jersey, Texas, Utah, and Vermont), 10 states experienced moderate ILI activity (Arizona, Colorado, Illinois, Indiana, Louisiana, Minnesota, North Dakota, Oregon, South Dakota, and Virginia), 13 states and the District of Columbia experienced low ILI activity (Arkansas, Florida, Georgia, Hawaii, Iowa, Massachusetts, Mississippi, Nebraska, New Mexico, New York, Oklahoma, Washington, and Wyoming), and 16 states experienced minimal ILI activity (Alaska, Connecticut, Delaware, Kentucky, Maine, Maryland, Montana, New Hampshire, North Carolina, Ohio, Pennsylvania, Rhode Island, South Carolina, Tennessee, West Virginia, and Wisconsin). As of February 9, 2013, the largest total number of jurisdictions experiencing high ILI activity in a single week occurred during the week ending December 29, 2012 (week 52), when a total of 33 states and New York City experienced high ILI activity. The total number of jurisdictions experiencing high ILI activity in a single week during the 2008–09 through 2011–12 influenza seasons has ranged from four to 18 jurisdictions, excluding the 2009 pandemic, when 44 jurisdictions reported high ILI activity (CDC, unpublished data, 2013).

## Geographic Spread of Influenza

For the week ending February 9, 2013 (week 6), the geographic spread of influenza†† was reported as widespread in 31 states (Alaska, Arizona, Arkansas, California, Colorado, Connecticut, Florida, Idaho, Illinois, Indiana, Iowa, Kansas, Maine, Maryland, Massachusetts, Michigan, Missouri, Montana, New Hampshire, New Jersey, New Mexico, New York, Ohio, Oklahoma, Oregon, Pennsylvania, Utah, Virginia, Washington, Wisconsin, and Wyoming), regional in Puerto Rico and 14 states (Alabama, Hawaii, Kentucky, Louisiana, Minnesota, Nebraska, Nevada, North Dakota, South Carolina, South Dakota, Tennessee, Texas, Vermont, and West Virginia), and local in the District of Columbia and four states (Georgia, Mississippi, North Carolina, and Rhode Island). Sporadic influenza activity was reported by Guam and one state (Delaware), and the U.S. Virgin Islands did not report. As of February 9, 2013, the number of jurisdictions reporting influenza activity as widespread peaked during the week ending January 12, 2013 (week 2), when a total of 48 jurisdictions reported influenza activity as widespread. The number of states reporting widespread activity during the peak week of activity has ranged from 25 to 49 states during the previous five influenza seasons (CDC, unpublished data, 2013).

## Influenza-Associated Hospitalizations

**FIGURE 3:**
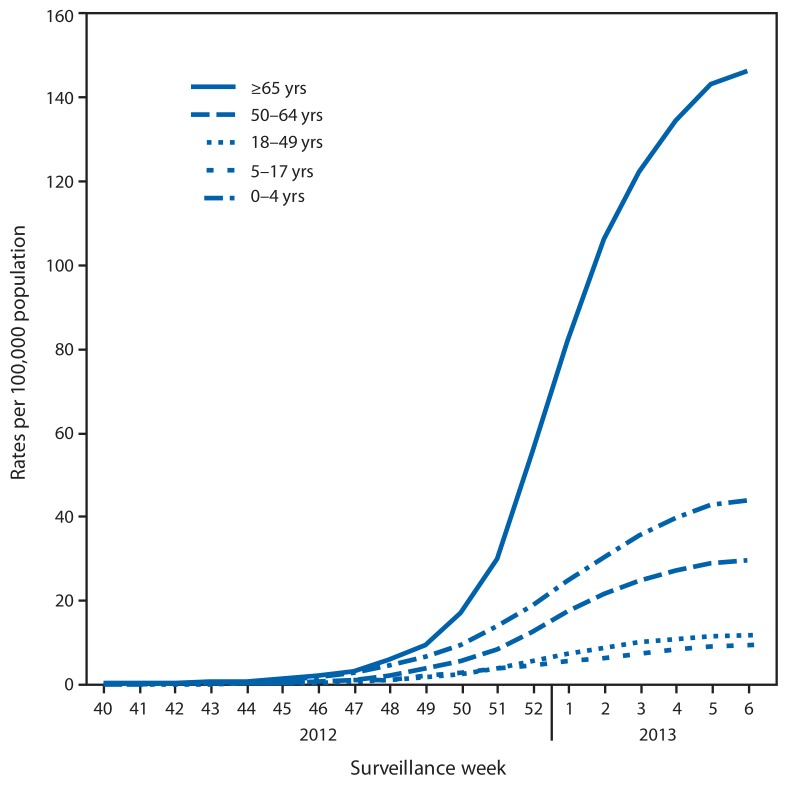
Rates of hospitalization for laboratory-confirmed influenza, by age group and surveillance week — FluSurv-NET,* 2012–13 influenza season^†^ * FluSurv-NET conducts population-based surveillance for laboratory-confirmed influenza-associated hospitalizations in children aged <18 years (since the 2003–04 influenza season) and adults aged ≥18 years (since the 2005–06 influenza season). The FluSurv-NET covers approximately 80 counties in the 10 Emerging Infections Program states (California, Colorado, Connecticut, Georgia, Maryland, Minnesota, New Mexico, New York, Oregon, and Tennessee) and additional Influenza Hospitalization Surveillance Project states (Iowa, Michigan, Ohio, Rhode Island, and Utah). ^†^ Data as of February 16, 2013.

CDC monitors hospitalizations associated with laboratory-confirmed influenza infection in adults and children through the Influenza Hospitalization Surveillance Network (FluSurv-NET),§§ which covers approximately 9% of the U.S. population. From October 1, 2012, to February 9, 2013, a total of 8,953 laboratory-confirmed influenza associated hospitalizations were reported, with a cumulative incidence for all age groups of 32.1 per 100,000 population. The most affected age group was persons aged ≥65 years, accounting for more than 50% of reported influenza-associated hospitalizations. The cumulative hospitalization rate (per 100,000 population) from October 1, 2012, to February 9, 2013, was 44.0 among children aged 0–4 years, 9.3 among children aged 5–17 years, 11.6 among adults 18–49 years, 29.4 among adults aged 50–64 years, and 146.2 among adults aged ≥65 years (Figure 3). During the past three influenza seasons (2009–10 through 2011–12), end-of-season age-specific cumulative hospitalization rates ranged from 14.8 to 73.0 per 100,000 population for ages 0–4 years, 4.0 to 27.3 for ages 5–17 years, 4.1 to 23.3 for ages 18–49 years, 8.3 to 30.4 for ages 50–64 years, and 25.3 to 64.0 for ages ≥65 years. During the 2005–06 to the 2008–09 influenza seasons, end-of-season hospitalization rates among adults aged ≥65 years ranged from 13.5 to 73.8 per 100,000 population.

For the current season, the most commonly reported underlying medical conditions among hospitalized adults were cardiovascular disease, metabolic disorders, obesity, and chronic lung disease (excluding asthma). The most commonly reported underlying medical conditions in hospitalized children were asthma, neurologic disorders, and immune suppression. Forty-four percent of hospitalized children had no identified underlying medical conditions that place them at higher risk for influenza complications.¶¶ Among 218 hospitalized women of childbearing age (15–44 years), 63 (29%) were pregnant.

## Pneumonia and Influenza-Associated Mortality

**FIGURE 4:**
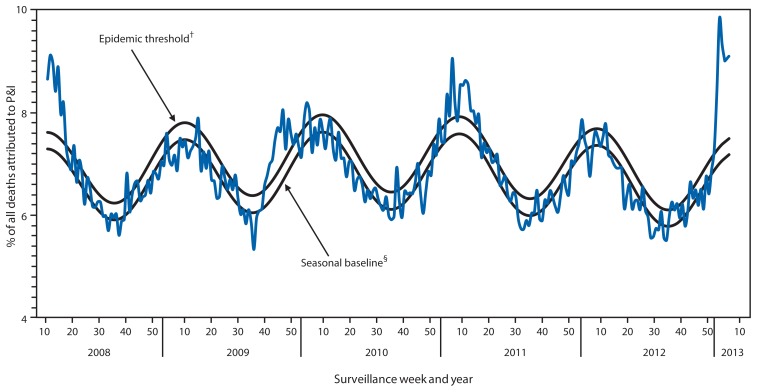
Percentage of all deaths attributable to pneumonia and influenza (P&I), by surveillance week and year — 122 U.S. Cities Mortality Reporting System, United States, 2008–2013* * For the reporting week ending February 9, 2013. ^†^ The epidemic threshold is 1.645 standard deviations above the seasonal baseline. ^§^ The seasonal baseline is projected using a robust regression procedure that applies a periodic regression model to the observed percentage of deaths from P&I during the preceding 5 years.

For the week ending February 9, 2013 (week 6), pneumonia and influenza (P&I) was reported as an underlying or contributing cause of death for 9.1% of all deaths reported to the 122 Cities Mortality Reporting System (Figure 4). This percentage is above the epidemic threshold of 7.5% for that week.*** Since September 30, 2012, the weekly percentage of deaths attributed to P&I ranged from 5.8% to 9.9%, and, as of February 9, 2013 (week 6), had exceeded the epidemic threshold for 6 consecutive weeks (weeks ending January 5–February 9, 2013 [weeks 1–6]). As of February 9, 2013, the weekly percentage of deaths attributed to P&I peaked at 9.9% during the week ending January 19, 2013 (week 3). Peak weekly percentages of deaths attributed to P&I in the previous five seasons ranged from 7.9% for the 2008–09 and 2011–12 seasons to 9.1% during the 2007–08 and 2010–11 seasons.

## Influenza-Associated Pediatric Mortality

As of February 9, 2013, a total of 64 laboratory-confirmed influenza-associated pediatric deaths occurring during the 2012–13 season had been reported to CDC from Chicago, New York City, and 27 states. The mean and median ages of children reported to have died were 7.9 and 7.4 years, respectively; three children were aged <6 months, 11 were aged 6–23 months, eight were aged 2–4 years, 24 were aged 5–11 years, and 18 were aged 12–17 years. Of the 64 deaths, 16 were associated with influenza A (H3N2) virus infection, 19 deaths were associated with an influenza A virus infection that was not subtyped, and 29 deaths were associated with influenza B infection. Since 2004, when CDC began collection of influenza-associated pediatric death data, each season approximately 20% of children aged ≥6 months who were eligible to receive seasonal influenza vaccination and died from influenza-associated complications had received the seasonal influenza vaccine (CDC, unpublished data, 2013). Since influenza-associated pediatric mortality became a nationally notifiable disease in 2004, the total number of influenza-associated pediatric deaths has ranged from 34 to 122 per season; excluding the 2009 pandemic, when 348 pediatric deaths were reported to CDC during April 15, 2009, through October 2, 2010.

### Editorial Note

The 2012–13 influenza season began early, and influenza activity remained elevated across the United States as of February 9, 2013; during the most recent weeks, decreases have been observed in the South and East, while increases have continued in the West. Although the timing of influenza activity is not predictable, substantial activity can occur as late as May (2). During September 30, 2012–February 9, 2013, influenza A (H3N2) viruses were identified most frequently, followed by influenza B viruses, but a small number of pH1N1 viruses also were reported. Antigenic characterization of influenza-positive respiratory specimens submitted to CDC indicated that the majority of these specimens were like the 2012–13 influenza vaccine components. As of February 9, 2013, more than half of influenza-associated hospitalizations were reported to have occurred in adults aged ≥65 years, and rates of influenza-associated hospitalization among adults aged ≥65 years increased sharply from late December through January. The weekly percentage of deaths attributed to P&I was above the epidemic threshold beginning early in January, with the majority of the P&I deaths occurring in adults aged ≥65 years.

What is already known on this topic?

CDC collects, compiles, and analyzes data on influenza activity year-round in the United States. The timing and severity of circulating influenza viruses can vary by geographic location and season.

What is added by this report?

Influenza activity in the United States began to increase in mid-November and remained elevated through February 9, 2013. During September 30, 2012–February 9, 2013, of 55,470 influenza viruses tested, 79% were influenza A, and 19% were influenza B. Of 29,914 influenza A viruses that were subtyped, 97% were H3N2, and 3% were pH1N1. The age group with the highest hospitalization rate was ≥65 years, accounting for more than half of all reported influenza-associated hospitalizations.

What are the implications for public health practice?

Year-round influenza surveillance provides critical information for planning interventions to prevent and control influenza, developing vaccine recommendations and antiviral treatment guidance, and presenting information to the public regarding the progress and severity of the influenza season.

In the past, higher overall and age-specific rates of hospitalization and mortality have been observed during influenza A (H3N2)–predominant seasons (3,4). Based on FluSurv-Net surveillance data for the 2012–13 season to date, rates of influenza-associated hospitalizations are highest among adults aged ≥65 years, followed by children aged 0–4 years. This trend is similar to that observed in the 2007–08 and 2010–11 influenza seasons, during which influenza A (H3N2) viruses predominated. The number and rate of influenza-associated hospitalizations among adults aged ≥65 years during the 2012–13 influenza season is the highest since data collection on laboratory-confirmed influenza-associated hospitalization in adults began in the 2005–06 season.

Vaccination remains the first and best way to prevent influenza and its complications. Health-care providers should continue to offer vaccine to all unvaccinated persons aged ≥6 months throughout the influenza season. Interim vaccine effectiveness estimates suggest that effectiveness against influenza A (H3N2) viruses is lower and not statistically significant in adults aged ≥65 years during the 2012–13 influenza season (5). Adults aged ≥65 years are at the greatest risk for hospitalization and death from influenza-associated complications; therefore, it is important for them to receive their annual influenza vaccine, take everyday preventive actions, and seek medical care quickly if they develop ILI symptoms to see if treatment with antiviral medications is needed. Antiviral medications remain an important adjunct to vaccination for reducing the health impact of influenza. Recommended antiviral medications are oseltamivir and zanamivir. Early and aggressive treatment with antiviral medication is crucial, ideally within the first 48 hours of illness onset, and persons with suspected influenza infection who are at high risk, including adults aged ≥65 years, should be treated with antiviral medications without the need to wait for laboratory confirmation of influenza (6). However, as indicated by observational studies, antiviral treatment might still be beneficial in patients with severe, complicated, or progressive illness and in hospitalized patients when started after 48 hours of illness onset (6). Recent data on influenza antiviral resistance indicate that >99% of currently circulating influenza virus strains are sensitive to these medications.

Influenza surveillance reports for the United States are posted online weekly and are available at http://www.cdc.gov/flu/weekly. Additional information regarding influenza viruses, influenza surveillance, influenza vaccine, influenza antiviral medications, and novel influenza A infections in humans is available at http://www.cdc.gov/flu.

## Footnotes

* The CDC influenza surveillance system collects five categories of information from eight data sources: 1) viral surveillance (U.S. World Health Organization collaborating laboratories, the National Respiratory and Enteric Virus Surveillance System, and novel influenza A virus case reporting); 2) outpatient illness surveillance (U.S. Outpatient Influenza-Like Illness Surveillance Network); 3) mortality (122 Cities Mortality Reporting System and influenza-associated pediatric mortality reports); 4) hospitalizations (FluSurv-NET, which includes the Emerging Infections Program and surveillance in five additional states); and 5) summary of the geographic spread of influenza (state and territorial epidemiologist reports).

† Defined as a temperature ≥100°F (≥37.8°C), oral or equivalent, and cough or sore throat, without a known cause other than influenza.

§ The national and regional baselines are the mean percentage of visits for ILI during noninfluenza weeks for the previous three seasons plus two standard deviations. Noninfluenza weeks are defined as periods of 2 or more consecutive weeks in which each week accounted for less than 2% of the season’s total number of specimens that tested positive for influenza. National and regional percentages of patient visits for ILI are weighted on the basis of state population. Use of the national baseline for regional data is not appropriate.

¶ The 10 regions include the following jurisdictions: *Region 1*: Connecticut, Maine, Massachusetts, New Hampshire, Rhode Island, and Vermont; *Region 2*: New Jersey, New York, Puerto Rico, and the U.S. Virgin Islands; *Region 3*: Delaware, District of Columbia, Maryland, Pennsylvania, Virginia, and West Virginia; *Region 4*: Alabama, Florida, Georgia, Kentucky, Mississippi, North Carolina, South Carolina, and Tennessee; *Region 5*: Illinois, Indiana, Michigan, Minnesota, Ohio, and Wisconsin; *Region 6*: Arkansas, Louisiana, New Mexico, Oklahoma, and Texas; *Region 7*: Iowa, Kansas, Missouri, and Nebraska; *Region 8*: Colorado, Montana, North Dakota, South Dakota, Utah, and Wyoming; *Region 9*: Arizona, California, Hawaii, Nevada, American Samoa, Commonwealth of the Northern Mariana Islands, Federated States of Micronesia, Guam, Marshall Islands, and Republic of Palau; and *Region 10*: Alaska, Idaho, Oregon, and Washington.

** Activity levels are based on the percentage of outpatient visits in a state attributed to ILI and are compared with the average percentage of ILI visits that occur during weeks with little influenza virus circulation. Activity levels range from minimal, which would correspond to ILI activity from outpatient clinics being at or below the average, to high, which would correspond to ILI activity from outpatient clinics being much higher than the average.

†† Levels of activity are 1) no activity; 2) sporadic: isolated laboratory-confirmed influenza cases or a laboratory-confirmed outbreak in one institution, with no increase in activity; 3) local: increased ILI, or at least two institutional outbreaks (ILI or laboratory-confirmed influenza) in one region of the state, with recent laboratory evidence of influenza in that region; virus activity no greater than sporadic in other regions; 4) regional: increased ILI activity or institutional outbreaks (ILI or laboratory-confirmed influenza) in at least two but less than half of the regions in the state with recent laboratory evidence of influenza in those regions; and 5) widespread: increased ILI activity or institutional outbreaks (ILI or laboratory-confirmed influenza) in at least half the regions in the state, with recent laboratory evidence of influenza in the state.

§§ FluSurv-NET conducts population-based surveillance for laboratory-confirmed influenza-associated hospitalizations in children aged <18 years (since the 2003–04 influenza season) and adults aged ≥18 years (since the 2005–06 influenza season). The FluSurv-NET covers approximately 80 counties in the 10 Emerging Infections Program states (California, Colorado, Connecticut, Georgia, Maryland, Minnesota, New Mexico, New York, Oregon, and Tennessee) and additional Influenza Hospitalization Surveillance Project (IHSP) states. IHSP began during the 2009–10 season to enhance surveillance during the 2009 H1N1 pandemic. IHSP sites included Iowa, Idaho, Michigan, Oklahoma, and South Dakota during the 2009–10 season; Idaho, Michigan, Ohio, Oklahoma, Rhode Island, and Utah during the 2010–11 season; Michigan, Ohio, Rhode Island, and Utah during the 2011–12 season; and Iowa, Michigan, Ohio, Rhode Island, and Utah during the 2012–13 season. Incidence rates are calculated using CDC’s National Center for Health Statistics population estimates for the counties included in the surveillance catchment area. Laboratory confirmation is dependent on clinician-ordered influenza testing, and testing for influenza often is underutilized because of the poor reliability of rapid test results and greater reliance on clinical diagnosis for influenza. As a consequence, cases identified as part of influenza hospitalization surveillance likely are an underestimation of the actual number of persons hospitalized with influenza.

¶¶ Persons at higher risk include children aged <5 years (especially those aged <2 years); adults aged ≥65 years; persons with chronic pulmonary (including asthma), cardiovascular (except hypertension alone), renal, hepatic, hematologic (including sickle cell disease), or metabolic disorders (including diabetes mellitus), or neurologic and neurodevelopment conditions (including disorders of the brain, spinal cord, peripheral nerve, and muscle, such as cerebral palsy, epilepsy [seizure disorders], stroke, intellectual disability [mental retardation], moderate to severe developmental delay, muscular dystrophy, or spinal cord injury); persons with immunosuppression, including that caused by medications or by human immunodeficiency virus infection; women who are pregnant or postpartum (within 2 weeks after delivery); persons aged ≤18 years who are receiving long-term aspirin therapy; American Indian/Alaska Natives; persons who are morbidly obese (i.e., body mass index ≥40); and residents of nursing homes and other chronic-care facilities.

*** The seasonal baseline proportion of P&I deaths is projected using a robust regression procedure in which a periodic regression model is applied to the observed percentage of deaths from P&I that were reported by the 122 Cities Mortality Reporting System during the preceding 5 years. The epidemic threshold is set at 1.645 standard deviations above the seasonal baseline.
